# Parameter estimation for multistage clonal expansion models from cancer incidence data: A practical identifiability analysis

**DOI:** 10.1371/journal.pcbi.1005431

**Published:** 2017-03-13

**Authors:** Andrew F. Brouwer, Rafael Meza, Marisa C. Eisenberg

**Affiliations:** Department of Epidemiology, University of Michigan, Ann Arbor, Michigan, United States of America; Johns Hopkins University, UNITED STATES

## Abstract

Many cancers are understood to be the product of multiple somatic mutations or other rate-limiting events. Multistage clonal expansion (MSCE) models are a class of continuous-time Markov chain models that capture the multi-hit initiation–promotion–malignant-conversion hypothesis of carcinogenesis. These models have been used broadly to investigate the epidemiology of many cancers, assess the impact of carcinogen exposures on cancer risk, and evaluate the potential impact of cancer prevention and control strategies on cancer rates. Structural identifiability (the analysis of the maximum parametric information available for a model given perfectly measured data) of certain MSCE models has been previously investigated. However, structural identifiability is a theoretical property and does not address the limitations of real data. In this study, we use pancreatic cancer as a case study to examine the practical identifiability of the two-, three-, and four-stage clonal expansion models given age-specific cancer incidence data using a numerical profile-likelihood approach. We demonstrate that, in the case of the three- and four-stage models, several parameters that are theoretically structurally identifiable, are, in practice, unidentifiable. This result means that key parameters such as the intermediate cell mutation rates are not individually identifiable from the data and that estimation of those parameters, even if structurally identifiable, will not be stable. We also show that products of these practically unidentifiable parameters are practically identifiable, and, based on this, we propose new reparameterizations of the model hazards that resolve the parameter estimation problems. Our results highlight the importance of identifiability to the interpretation of model parameter estimates.

## Author summary

Parameter estimation from data is an important part of mathematical modeling, and structural identifiability is the study of what parametric information exists, for a given model, in ideal data. Unfortunately, for a variety of reasons, there is often less information available in our real data sets. The study of these problems is called practical identifiability. In this study, we consider a family of models of cancer biology that are commonly used to explain cancer incidence in terms of underlying biological parameters. Using profile likelihoods, a widely applicable numerical tool, we demonstrate that even though the more complex models we consider have theoretically more identifiable parameters, the data contains only three pieces of practically identifiable information for each model: the product of the initiating mutation rates, the net cell proliferation rate, and the scaled malignant conversion rate. This result can be interpreted biologically: we can determine only the product of cell mutation rates not the intermediate rates themselves. Our result limits the interpretability of previous work, but we propose a novel parameterization to resolve the identifiability issue. Ultimately, our analysis demonstrates the importance of verifying the practical identifiability of parameters before assigning too much weight to the interpretation of their estimated values.

## Introduction

Parameter estimation is an important aspect of computational modeling in the life sciences because parameter estimates can shed light on underlying biological mechanisms and processes and provide a way to link dynamic models to real-world data. However, the dynamics of many living systems have evolved to be robust to changes in underlying parameters, which necessitates an understanding of which parameters or combinations of parameters can even be estimated from data, known as identifiability. Here, we leverage computational identifiability tools to determine what cancer incidence data can tell us about the biology of carcinogenesis.

Cancers arise from the accumulation of genetic (and epigenetic) abnormalities and mutations. Although a single change is thought to be sufficient for certain cancers (certain leukemias, lymphomas, and sarcomas in particular), many cancers are thought to require two or more hits [1]. For example, retinoblastoma is a two-hit cancer—indeed, a two-hit model of retinoblastoma predicted the existence of the tumor suppressing gene pRb before it was discovered [2]—and colorectal cancer can be described by three or more hits to the APC, RAS, and P53 genes [1]. Similarly to the development of precancerous polyps for colorectal cancer, many esophageal cancers begin with a transition to a condition called Barrett’s esophagous [3] before accumulating additional abnormalities. These genetic (or epigenetic) hits are often described as starting different phases of carcinogenesis: initiation, the first destabilizing mutation(s); promotion, the unchecked growth of a tumor; and malignant conversion, the spread into other tissues. This classification is useful because different exposures may act on different stages of carcinogenesis.

Multistage clonal expansion (MSCE) models are a class of continuous-time Markov chain models that capture this initiation–promotion–malignant-conversion hypothesis of carcinogenesis. Originally posed as a two-stage model [4, 5] using birth–death–mutation branching-process theory, this class of models has been expanded to three or more stages, multiple pathways, and other variations. These models have been successfully used to analyze epidemiological population-level cancer incidence data [6–11], to assess the impact of time-varying exposures on cancer risk using individual-level data [12–16], and to project the impact of prevention and control strategies on population cancer rates [10, 17–19]. Although models that use multiple clonal expansion steps have been considered, models with multiple initiation stages but only a single, final clonal expansion stage are more common in the literature and appear to capture the incidence patterns of many cancers (e.g. [6–8, 20]. We are concerned here with parameter estimation for MSCE models because it can lead to better understanding of the rates of biological processes like tumor growth or adverse mutations. Indeed, knowing the approximate speed at which an abnormality arises may help to classify the underlying abnormal event (e.g. single nucleotide mutation, chromosomal translocation, or epigenetic change).

Identifiability is the study of the parametric information available in a data set when viewed through the lens of a model, and identifiability analysis is an important precursor to accurate parameter estimation. A model is said to be *identifiable* if all model parameters may be uniquely determined from observed data [21–23]. There are two kinds of identifiability analyses: structural—which analyzes the model in the context of perfectly measured and noise-free data in order to uncover the inherent limitations of the model structure in the context of parameter estimation—and practical—which considers obstacles to parameter estimation that arise from noise, sampling frequency, bias, and other issues in real-world data sets [24]. Identifiability analysis can identify parameter combinations that embody the parametric information available in the data and lead to useful reparameterizations of the model [23].

That MSCE models are not fully identifiable is well established [6, 25–27]. In particular, finding the closed-form solution of a model’s hazard function—the model output corresponding to age-specific incidence data—gives an upper bound on the number of identifiable parameter combinations available for that model from the age-specific incidence data and constrains the forms of those combinations. We previously computed the exact structural identifiability for the class of MSCE models with constant parameters and one clonal-expansion step [28]. However, this is not the last word on the identifiability of MSCE models. In particular, it is known that there is a practical identifiability problem with the clonal expansion models with three or more stages: the information contained in the asymptote of the corresponding hazard function is not available in the usual age-specific cancer incidence data because the asymptote is not reached within human lifespans [8].

In this analysis, we examine this practical identifiability problem with a profile-likelihood approach. We consider pancreatic cancer, which has linear age-specific incidence at older ages [8] and can be fit by an MSCE model with two or more stages. We demonstrate that the two-, three-, and four-stage models have only three practically identifiable parameter combinations and that, for the three- and four-stage models, several parameters that are theoretically structurally identifiable individually, are, practically, identifiable only in their product. This practical unidentifiability means the incidence data contains information about the overall rate of progression from normal to cancer-initiated cells but not the expected information on the rates of the individual steps leading to initiation.

## Methods

### Multistage clonal expansion models

**Fig 1:**
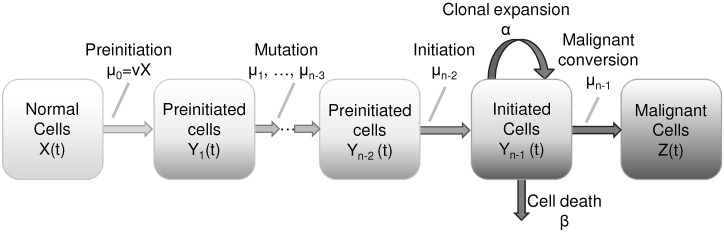
Schematic of a general multistage clonal expansion model. Multistage clonal expansion models are continuous-time Markov chain models in which normal cells undergo a series of genetic changes that lead to a state of clonal expansion followed by progression to malignancy.

The mathematics of multistage clonal expansion models have been detailed elsewhere [4, 5, 8, 25, 29–36], so we only give a basic description here. The *n*-stage clonal expansion model (Fig 1) is a continuous-time Markov chain with the following states: *X*(*t*), the number of normal cells at age *t*; *Y*_1_(*t*), …, *Y*_*n*−2_, the number of cells in subsequent preintiation states; *Y*_*n*−1_(*t*), the number of initiated cells; and *Z*(*t*), the number of malignant cells. Let *ν* be the initial mutation rate, *μ*_1_, …, *μ*_*n*−3_ the following preinitiation mutation rates, *μ*_*n*−2_ the initiation mutation rate, *μ*_*n*−1_ the malignant transformation rate, *α* the clonal expansion rate, and *β* the cell death rate. If the parameters and *X*(*t*) are constant, then we may denote

$$p_{n},q_{n}≔\frac{1}{2}\;\left(-\left(\alpha -\beta -\mu_{n-1}\right)\mp \sqrt{{\left(\alpha -\beta -\mu_{n-1}\right)}^{2}+4\alpha \mu_{n-1}}\right),$$ (1)

and write hazard functions [6, 8] of the two-, three-, and four-stage models (a derivation is provided in S1 Text):

$$h_{2}\left(t\right)=\frac{\nu X}{\alpha }\;\left(\frac{p_{2}q_{2}\left(e^{-q_{2}t}-e^{-p_{2}t}\right)}{q_{2}e^{-p_{2}t}-p_{2}e^{-q_{2}t}}\right),$$ (2)

$$h_{3}\left(t\right)=\nu X\;\left(1-\;{\left(\frac{q_{3}-p_{3}}{q_{3}e^{-p_{3}t}-p_{3}e^{-q_{3}t}}\right)}^{\mu_{1}/\alpha }\right),$$ (3)

$$h_{4}\left(t\right)=\nu X\;\left(1-\exp\;\left(\int_{0}^{t}\mu_{1}\;\left({\left(\frac{q_{4}-p_{4}}{q_{4}e^{-p_{4}\left(t-u\right)}-p_{4}e^{-q_{4}\left(t-u\right)}}\right)}^{\mu_{2}/\alpha }-1\right)\;du\right)\right).$$ (4)

From the hazard functions, we can see that the two-, three-, and four-stage models have at most three (*νX*/*α*, *p*_2_, *q*_2_), four (*νX*, *μ*_1_/*α*, *p*_3_, *q*_3_), and five (*νX*, *μ*_1_, *μ*_2_/*α*, *p*_4_, *q*_4_) structurally identifiable parameter combinations. In this case, these parameter combinations are structurally identifiable [28].

**Fig 2:**
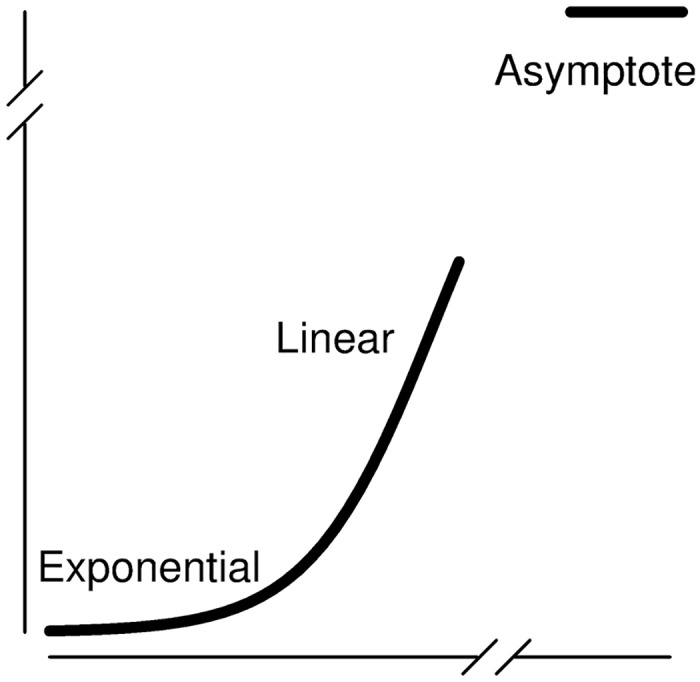
Salient features of a multistage clonal expansion model hazard. Multistage clonal expansion models have exponential and linear phases that may be observed on the time scale of a human lifespan. Depending on the number of stages in the model, the asymptote may or may not occur within a human lifespan.

Multistage clonal expansion model hazards share similar characteristics, including an exponential region, a linear region, and an asymptote (Fig 2). The transition from the linear phase to the asymptote occurs on different time scales for the different models, and, for biologically reasonable ranges of the parameters, only *h*_2_ can achieve this asymptote within human lifespans. The other hazards achieve their asymptotes on the order of 1,000 to 100,000 years, depending on the parameters. For example, the asymptote of the three-stage model occurs on the order of (*μ*_1_(1 − *β*/*α*))^−1^ [8], and mutation rate estimates are typically on the order of 10^−7^–10^−5^ [4–8] (note that 0 < (1 − *β*/*α*) < 1, so that this term can only exacerbate the time span). Thus, because real data cannot access the information contained in the asymptote and other late appearing features, one may expect inherent practical identification issues for MSCE models with more stages.

### Data

We consider cancers reported to the Surveillance, Epidemiology, and End Results (SEER) cancer registries, using SEER 9 data 1973–2012 (data available in S1 and S2 Data). We use the International Classification of Diseases (ICD-10) codes to identify incidence of pancreatic cancer (C25).

### Identifiability framework

More thorough treatments of identifiability of dynamical systems are presented elsewhere [23, 24, 37, 38], and we previously described a framework to apply dynamical systems identifiability techniques to stochastic time-to-event models, including multistage clonal expansion models [28]. Nevertheless, we provide the basic identifiability framework and definitions here for reference.

Consider a vector of states ***x***(*t*) (unobserved), vector of parameters to be estimated ***ρ***, and observed (known) input *u*(*t*) and output *v*(*t*) in the dynamical systems model,

$$\begin{array}{cc} \dot{x}\left(t\right) & =f(x(t),u(t),\rho ),\\ v(t) & =g(x(t),\rho ). \end{array}$$ (5)

**Definition 1** *Parameter* *ρ*_*i*_ *in the model given in* Eq (5) *is (globally) structurally identifiable if, for almost all values* $\rho_{i}^{*}$ *and initial conditions, the observation of an output trajectory (v*(*t*) = v*(*t*)*)* *uniquely identifies* *ρ*_*i*_ *(*$\rho_{i}=\rho_{i}^{*}$), i.e. if only one value of *ρ*_*i*_ *could have resulted in the observed output*.

**Definition 2** *The model given in* Eq (5) *is (globally) structurally identifiable if each* *ρ*_*i*_ *is structurally identifiable*.

The definition of structural identifiability concerns perfectly measured input and output. However, because real data may not capture all of the parameteric information available in a theoretic trajectory, parameters that are structurally identifiable in a model for a kind of theoretical data may be practically unidentifiable given a corresponding real-world dataset. Practical non-identifiability can arise from poor data quality (uncertainty, infrequent sampling, etc), but it can also be inherent to the type of data measured. For example, the saturation constant of a Michaelis-Menten equation may not be identifiable from low-dose data [39], and the amplitude of a circadian rhythm will not be identifiable if a value is measured once a day at the same time [40]. Thus, even if there are a large number of data points (e.g. as is often the case for cancer registry data), practical identifiability may still be an issue. It is this kind of inherent limitation of the data that we explore for the multistage clonal expansion models.

Practical identifiability is difficult to define in a rigorous way without choosing a threshold (e.g. width of a confidence interval) and thus has a “I know it when I see it” quality. Nevertheless, descriptions of practical identifiability are possible and typically consider the confidence bounds for the estimated parameters, found by Fisher Information Matrix (FIM) [22, 23, 41, 42] or likelihood-based methods [24]. In this analysis, we use likelihood-based confidence intervals, which are defined as follows. Let L(ρ) be the likelihood for the model given the data set as a function of the parameters ***ρ***, and let $\hat{\rho }$ the maximum-likelihood estimator.

**Definition 3** *Let* $L^{*}\left(\rho_{i}\right)$ *denote the maximum likelihood when the* *ith parameter is fixed to value* *ρ_i_, and call it the* *profile likelihood* *of* *ρ_i_. Then, the* *likelihood-based confidence interval* *for* *ρ*_*i*_ *at level of significance* *α* *is the set of values of* *ρ*_*i*_ *for which the relative negative log-likelihood at* *ρ*_*i*_ *is less than a threshold determined by* *α, that is,*

$$\{\rho_{i}\;:\log\left(L\left(\hat{\rho }\right)\right)-\log\left(L^{*}\left(\rho_{i}\right)\right)<\Delta_{\alpha }\},$$ (6)

*where*

$$2\Delta_{\alpha }=\chi^{2}\left(\alpha ,\text{df}\right)$$ (7)

*is the chi-squared distribution with a number of degrees of freedom (df) equal to the number of parameters (for simultaneous confidence intervals) or equal to 1 (for pointwise confidence intervals).* [24, 43].

We would like to say that parameter *ρ*_*i*_ in the model given in Eq (5) is practically identifiable if the likelihood-based confidence interval for *ρ*_*i*_ has finite length. However, this definition is neither well-defined (the confidence interval may be finite for one level of significance but infinite at another) nor practically verifiable. Ultimately, parameters with confidence intervals that are sufficiently large—typically orders of magnitude—as to cause uncertainty and parameter estimation problems at the desired parameter scale and level of significance can be said to be *practically unidentifiable*.

### Computation methods

We use profile likelihood [24] and subset profiling [42] methods to investigate the practical identifiability of the two-, three-, and four-stage models. We assume that cancer incidence is Poisson distributed (details in S1 Text). Profile likelihoods were computed by fixing the value of one parameter at each of a series of values within an interval and numerically optimizing the negative log-likelihood as a function of the remaining parameters. Numerical optimization was done in R (v.3.0.1) using the Bhat package [44].

## Results

**Fig 3:**
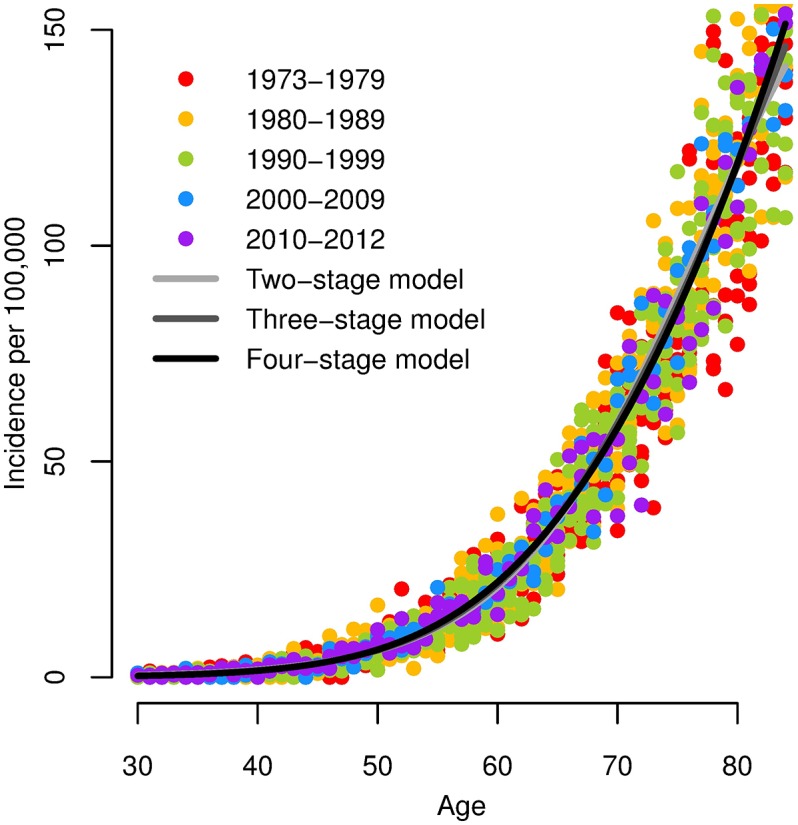
Pancreatic cancer incidence and best-fit MSCE models. Incidence of pancreatic cancer in men per 100,000 (SEER 9, 1973–2012), and best-fit two-, three-, and four-stage clonal expansion model hazards. Note that the three model hazards largely overlap.

We plot incidence rates of pancreatic cancer reported to SEER 9 (1973–2012) in men by decade (Fig 3). The data exhibit the classic pattern of linear incidence at older ages. There are no apparent temporal trends, so we fit the two-, three-, and four-stage clonal expansion model hazards to the entire data set by minimizing the negative log-likelihood. The Akaike Information Criterion (AIC) for each model (relative to the best-fitting model) is 177.7, 72.3, and 0, respectively, which preferences the four-stage model.

### Two-stage model

**Fig 4:**
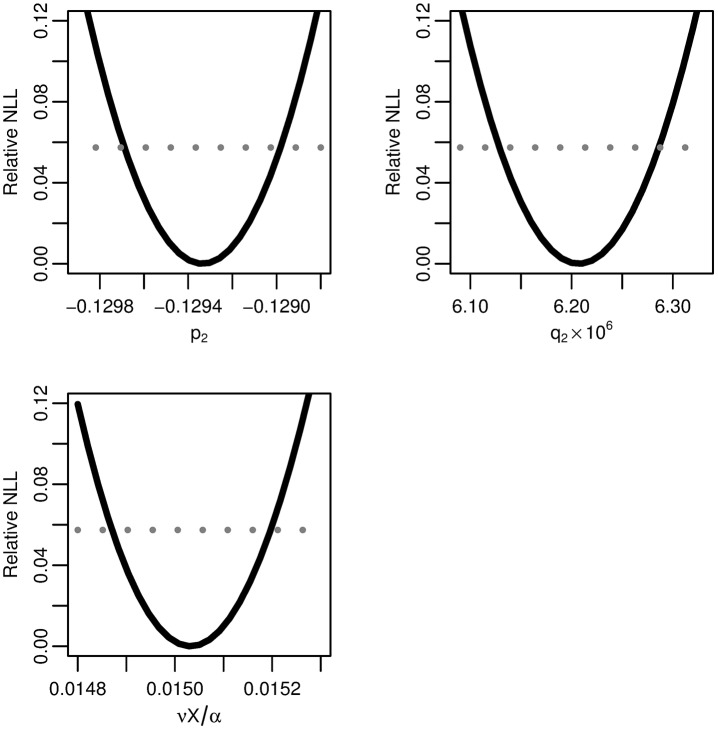
Two-stage model profile likelihoods. Profiles of the relative negative log-likelihood (NLL) of the two-stage clonal expansion model as each of the parameter combinations *p*_2_, *q*_2_, and *νX*/*α* are varied while the remaining parameters are fit. The gray dotted line gives the *α* = 0.01 threshold for simultaneous confidence intervals based on the relative negative log-likelihood. All three parameters are identifiable.

We profile the relative negative log-likelihood of the maximum-likelihood two-stage hazard as a function of each of the parameter combinations *p*_3_, *q*_3_, and *νX*/*α* (Fig 4). All three parameters combinations are practically identifiable because of the trough-shape of the negative log-likelihood, giving finite confidence intervals. The parameter estimates are given in Table 1.

**Table 1:** Best-fit parameters and likelihood-based 99% confidence intervals. Best-fit parameters and likelihood-based 99% confidence intervals for the fits of the two-, three-, and four-stage clonal expansion models (with parameterizations using only practically identifiable parameter combinations and given in Eqs (2), (9) and (11) respectively) to age-specific incidence of pancreatic cancer.

Model	Parameter combination	Value	Likelihood-based 99% CI
**Two-stage**			
	$p_{2}=\frac{1}{2}\;(-\;(\alpha -\beta -\mu_{1})-\sqrt{(\alpha -\beta -\mu_{1})+4\alpha \mu_{1}})$	-1.29E-1	(-1.30E-1, -1.29E-1)
	$q_{2}=\frac{1}{2}\;(-\;(\alpha -\beta -\mu_{1})+\sqrt{(\alpha -\beta -\mu_{1})+4\alpha \mu_{1}})$	6.21E-6	(6.13E-6, 6.29E-6)
	*r*_2_ = *νX*/*α*	1.50E-2	(1.49E-2, 1.52E-2)
**Three-stage**			
	$p_{3}=\frac{1}{2}\;(-\;(\alpha -\beta -\mu_{2})-\sqrt{(\alpha -\beta -\mu_{2})+4\alpha \mu_{2}})$	-1.38E-1	(-1.39E-1, -1.37E-1)
	$q_{3}=\frac{1}{2}\;(-\;(\alpha -\beta -\mu_{2})+\sqrt{(\alpha -\beta -\mu_{2})+4\alpha \mu_{2}})$	1.57E-5	(1.53E-5, 1.60E-5)
	$r_{3}=\sqrt{\nu X\mu_{1}/\alpha }$	2.35E-2	(2.33E-2, 2.38E-2)
**Four-stage**			
	$p_{4}=\frac{1}{2}\;(-\;(\alpha -\beta -\mu_{3})-\sqrt{(\alpha -\beta -\mu_{3})+4\alpha \mu_{3}})$	-1.50E-1	(-1.52E-1, -1.48E-1)
	$q_{4}=\frac{1}{2}\;(-\;(\alpha -\beta -\mu_{3})+\sqrt{(\alpha -\beta -\mu_{3})+4\alpha \mu_{3}})$	4.59E-5	(4.40E-5, 4.78E-5)
	*r*_4_ = (*νXμ*_1_*μ*_2_/*α*)^1/3^	2.66E-2	(2.63E-2, 2.70E-2)

### Three-stage model

**Fig 5:**
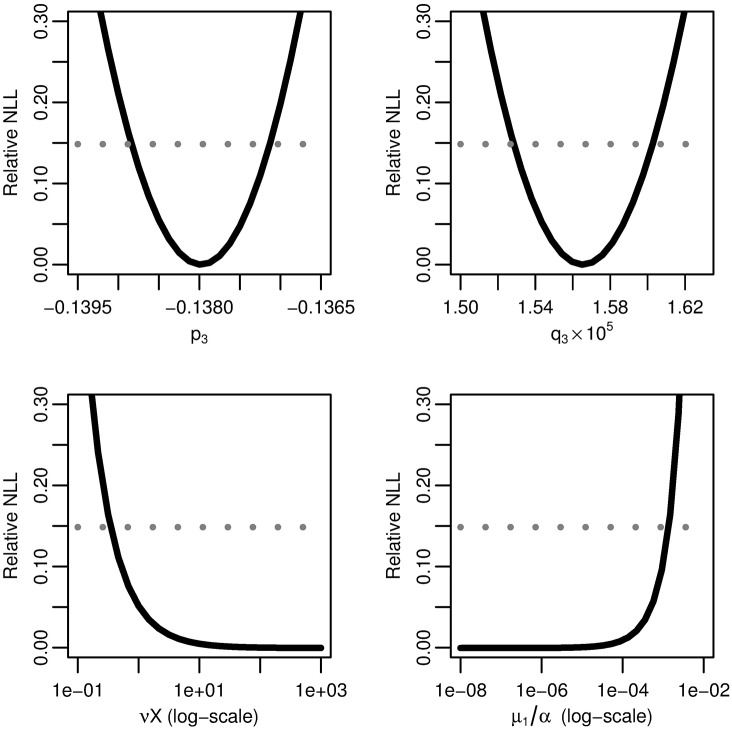
Three-stage model profile likelihoods. Profiles of the relative negative log-likelihood (NLL) of the three-stage clonal expansion model as each of parameter combinations *p*_3_, *q*_3_, *νX*, and *μ*_1_/*α* are varied while the remaining parameters are fit. The gray dotted line gives the *α* = 0.01 threshold for simultaneous confidence intervals based on the relative negative log-likelihood. Parameter combinations *p*_3_ and *p*_4_ are identifiable, while *νX* and *μ*_1_/*α* are practically unidentifiable.

We profile the relative negative log-likelihood of the maximum-likelihood three-stage hazard as a function of each of the parameter combinations *p*_3_, *q*_3_, *νX*, and *μ*_1_/*α* (Fig 5). Parameter combinations *p*_3_ and *q*_3_ are practically identifiable as above, but parameter combinations *νX* and *μ*_1_/*α* are not practically identifiable because their likelihoods flatten out, resulting in infinite confidence intervals.

**Fig 6:**
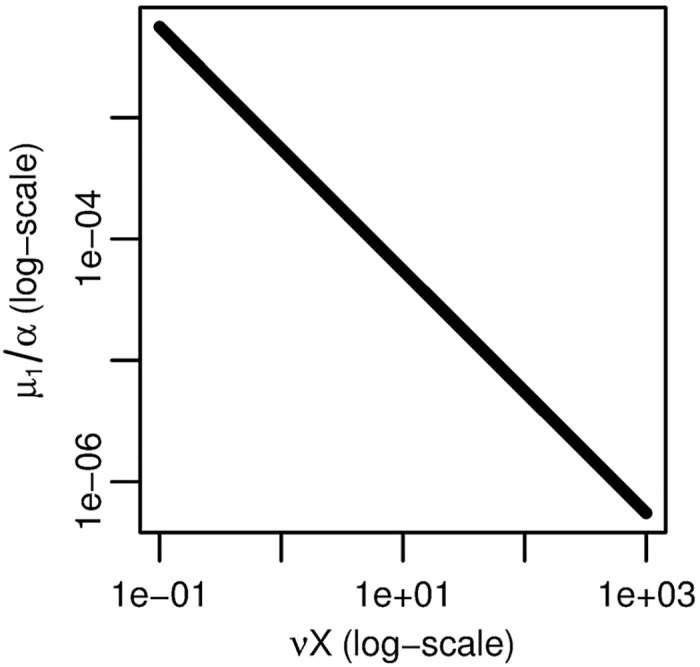
Three-stage model parameter dependency. Fitted value of *μ*_1_/*α* as *νX* is varied for the three-stage clonal expansion model. The linear relationship on a log–log scale indicates an identifiable product.

To identify the form of the practically-identifiable parameter combination of *νX* and *μ*_1_/*α*, we plot the fitted value of *μ*_1_/*α* as we vary the value of *νX* (Fig 6). Because the relationship is linear on the log–log scale, *νX* and *μ*_1_/*α* exist in a practically identifiable product. From the biological perspective, this means that we can only know the net rate of transition from normal to initiated cells but not the rates of the individual intermediate steps.

Our analysis thus demonstrates that there are three parameter combinations that are practically identifiable for the three-stage model from age-specific cancer-incidence data. Since there are three pieces of information in the data and four degrees of freedom in the full model (Eq 3), one might assume that one additional constraint on the model is sufficient to reduce the number of parameters estimated to three and simultaneously resolve the non-identifiability problem. However, the most reasonable simplifying assumption, namely that the first two mutation rates are the same (*ν* = *μ*_1_), such as for biallelic gene inactivation [1], does not do this; the three-stage model with *ν* = *μ*_1_ still has four structurally identifiable parameter combinations, namely *p*_3_, *q*_3_, *νX*, and *ν*/*α*, but only three pieces of practically identifiable information, so another constraint would be needed for a fully identifiable model. In this case, the constraint would need to designate the relative values of *νX* and *μ*_1_/*α*, which assuming *ν* = *μ*_1_ does not do. The *ν* = *μ*_1_ assumption does, however, suggest a new reparameterization of Eq 3. Denote

$$r_{3}≔\sqrt{\left(\nu X\right)\left(\mu_{1}/\alpha \right)},$$ (8)

and fix *X* and *α* at reasonable values, i.e. at values where the likelihood profiles are flat (see Fig 5). Then, assuming *ν* = *μ*_1_, we parameterize $\nu X=r_{3}\sqrt{\alpha X}$ and $\mu_{1}/\alpha =r_{3}/\sqrt{\alpha X}$, and write

$$h_{3}\left(t\right)=r_{3}\sqrt{\alpha X}\;\left(1-\;{\left(\frac{q_{3}-p_{3}}{q_{3}e^{-p_{3}t}-p_{3}e^{-q_{3}t}}\right)}^{r_{3}/\sqrt{\alpha X}}\right).$$ (9)

As long as *X* and *α* are chosen so that *νX* and *μ*_1_/*α* are within a the range of values for which the likelihood is flat, their exact values do not affect the model fit and can be fixed. Caution is advisable here, however: although the exact values of these parameters do not affect the fit in this context, it is important to not take these values into other contexts where the exact values may be relevant, e.g. prediction in context of time-varying exposures. Nevertheless, this parameterization has several advantages. In particular, multiplicative effects on *r*_3_, such as relative period or cohort effects, can be thought of as affecting both *ν* and *μ*_1_ equally: under the assumption *μ* ≔ *ν* = *μ*_1_, *r*_3_ simplifies to $r_{3}=\mu \sqrt{X/\alpha }$, and, more generally, we can write, for some scalar *ξ*, $\xi r_{3}=\sqrt{\left(\xi \nu \right)\left(\xi \mu_{1}\right)\left(X/\alpha \right)}$.

**Fig 7:**
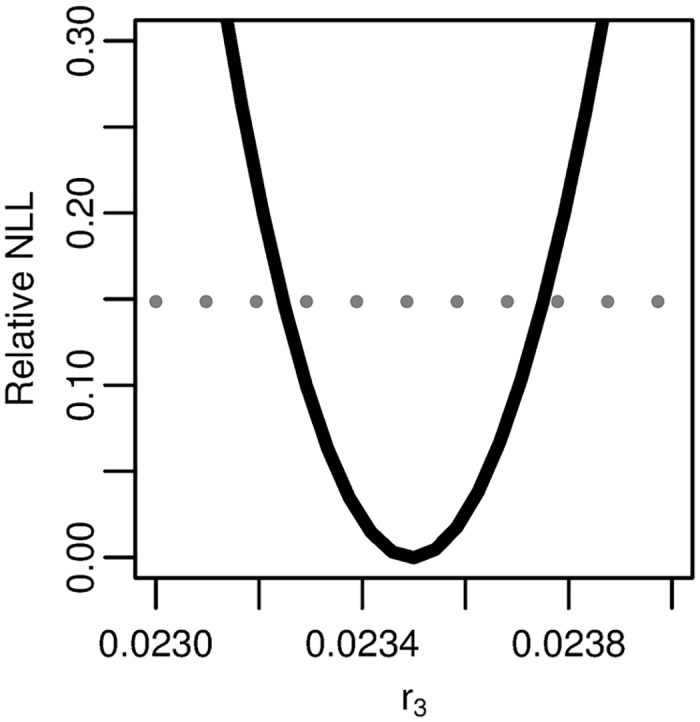
Profile likelihood for the reparameterized combination of the three-stage model. Profile of the relative negative log-likelihood (NLL) as the parameter $r_{3}=\sqrt{\nu X\mu_{1}/\alpha }$ is varied while the remaining parameters are fit in the three-stage clonal expansion model. The gray dotted line gives the *α* = 0.01 threshold for simultaneous confidence intervals based on the relative negative log-likelihood. Parameter combination *r*_3_ is identifiable.

We see that the profile relative NLL of $r_{3}=\sqrt{\nu \mu_{1}X/\alpha }$ has a finite confidence interval (Fig 7), as *p*_3_ and *q*_3_ did in Fig 5. The best-fit parameters for the three-stage model—parameterized as in Eq (9) and fit to the age-specific pancreatic cancer incidence data—are given in Table 1.

### Four-stage model

**Fig 8:**
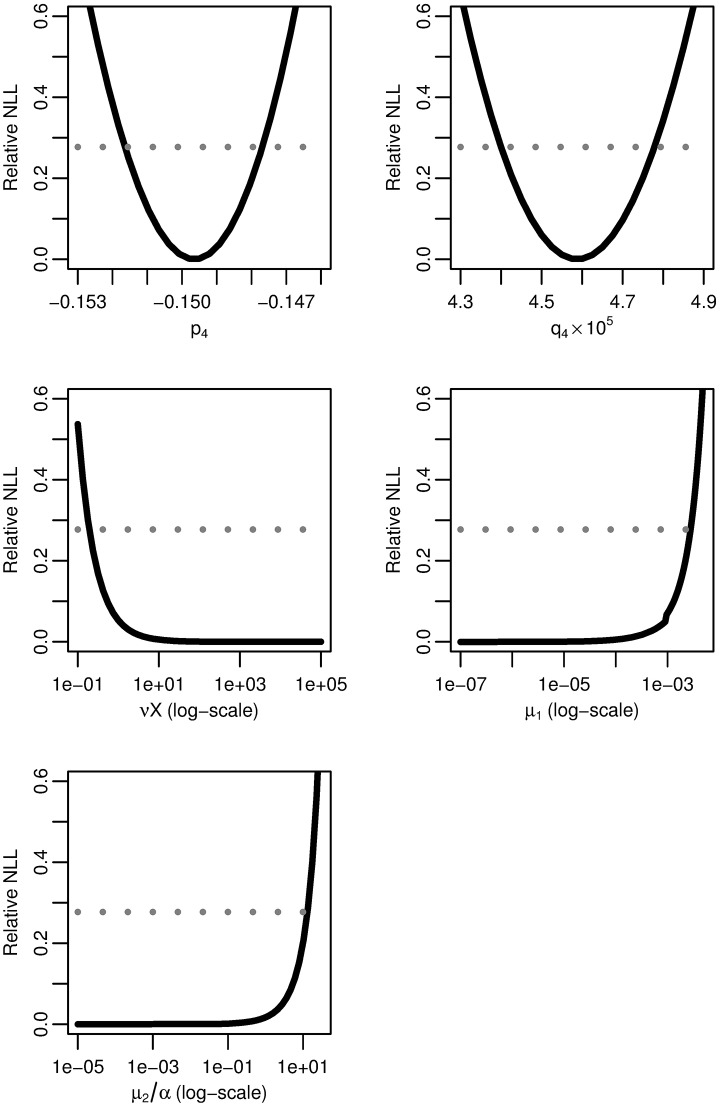
Four-stage model profile likelihoods. Profiles of the relative negative log-likelihood (NLL) of the four-stage clonal expansion model as each of parameter combinations *p*_4_, *q*_4_, *νX*, *μ*_1_, and *μ*_2_/*α* are varied while the remaining parameters are fit. The gray dotted line gives the *α* = 0.01 threshold for simultaneous confidence intervals based on the relative negative log-likelihood. Parameter combinations *p*_4_ and *q*_4_ are identifiable, while *νX*, *μ*_1_, and *μ*_2_/*α* are practically unidentifiable.

We similarly profile the relative negative log-likelihood of the maximum-likelihood four-stage hazard as a function of each of the parameter combinations *p*_4_, *q*_4_, *νX*, *μ*_1_, and *μ*_2_/*α* (Fig 8). As before, parameters combinations *p*_4_ and *q*_4_ have finite confidence intervals and are practically identifiable, while combinations *νX*, *μ*_1_ and *μ*_2_/*α* have infinite confidence intervals and are not practically identifiable.

**Fig 9:**
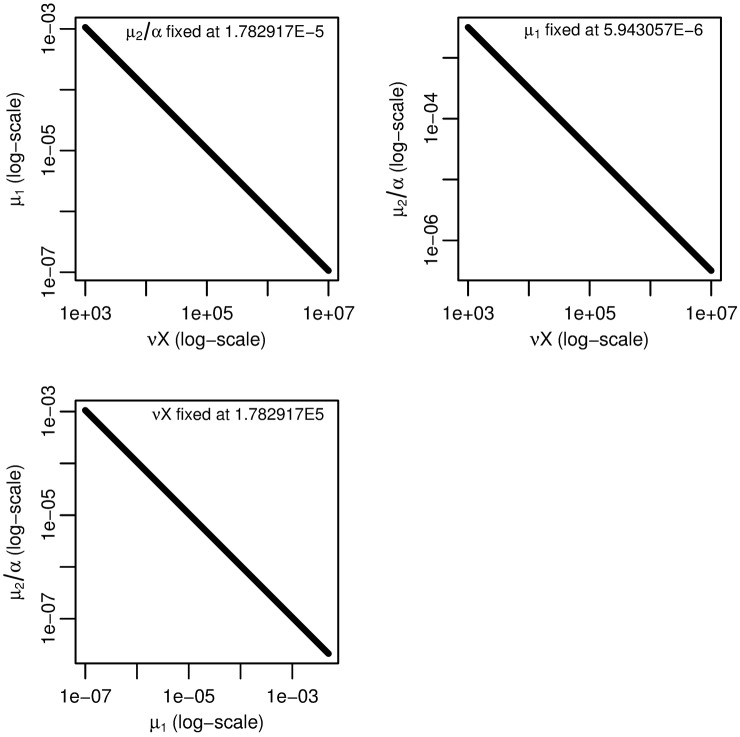
Four-stage model parameter dependencies. Fitted values of one of *νX*, *μ*_1_, or *μ*_2_/*α* as another is fixed and the third is varied for the four-stage model. The linear relationships on a log–log scale indicate that *νXμ*_1_*μ*_2_/*α* is an identifiable product.

To determine the combination structure, we use subset profiling [42]. However, rather than using FIM to determine the profiled parameter subsets, we note that the analysis of the three stage model leads us to suspect that the three parameter combinations *νX*, *μ*_1_, and *μ*_2_/*α* are in a practical product. We use this structure to propose our nearly-full rank subsets. To verify this proposal, we plot the fitted value of one parameter combination while another is fixed and the third is varied (Fig 9). The three selected plots presented are sufficient to verify that the three parameter combinations indeed exist in a practically identifiable product. As for the three-stage case, that *νX*, *μ*_1_, and *μ*_2_/*α* can only be identified up to their product means that we can only know the net rate of transition from normal to initiated cells but not the rates of the individual intermediate steps.

We can define a quantity analogous to *r*_3_ in the three stage case. Here,

$$r_{4}={\left(\nu X\mu_{1}\mu_{2}/\alpha \right)}^{1/3}$$ (10)

and, for some reasonable fixed values of *X* and *α*,

$$h_{4}\left(t\right)=r_{4}\;{\left(X^{2}\alpha \right)}^{1/3}\left(1-\exp\;\left(\int_{0}^{t}r_{4}{\left(\alpha /X\right)}^{1/3}\;\left({\left(\frac{q_{4}-p_{4}}{q_{4}e^{-p_{4}\left(t-u\right)}-p_{4}e^{-q_{4}\left(t-u\right)}}\right)}^{r_{4}/{\left(\alpha^{2}X\right)}^{1/3}}-1\right)\;du\right)\right).$$ (11)

**Fig 10:**
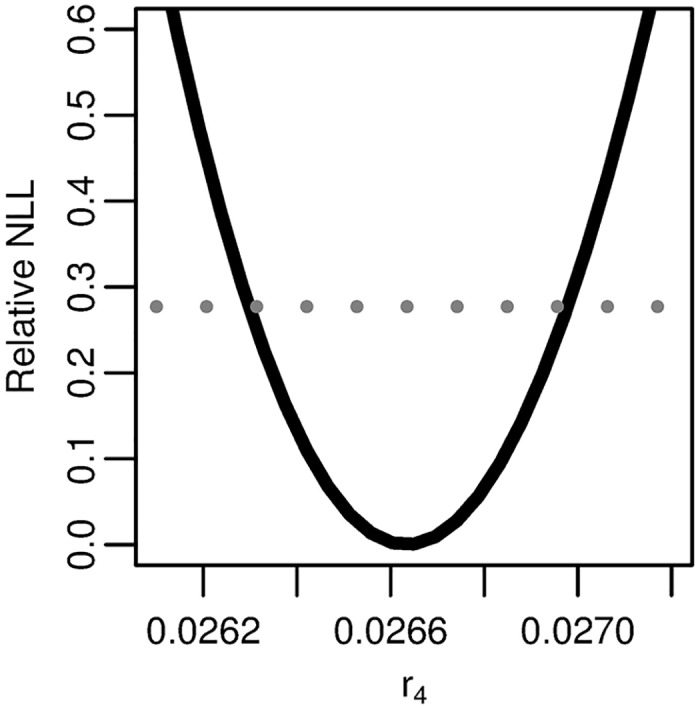
Profile likelihood for the reparameterized combination of the four-stage model. Profile of the relative negative log-likelihood (NLL) as the parameter *r*_4_ = (*νXμ*_1_*μ*_2_/*α*)^1/3^ is varied while the remaining parameters are fit in the four stage clonal expansion model. The gray dotted line gives the *α* = 0.01 threshold for simultaneous confidence intervals based on the relative negative log-likelihood. Parameter combination *r*_4_ is identifiable.

We see that the profile relative NLL of *r*_4_ = (*νXμ*_1_*μ*_2_/*α*)^1/3^ has the expected trough shape (Fig 10), as seen in Fig 8 for *p*_4_ and *q*_4_. The best-fit parameters for the four-stage model—parameterized as in Eq (11) and fit to the age-specific pancreatic cancer incidence data—are given in Table 1.

## Discussion

Practical unidentifiability is a significant barrier to parameter estimation. Indeed, because it—unlike structural identifiability—can be so dependent on the quality of the data, it can be a moving target. From this perspective, ironically, it is perhaps fortunate that the practical identifiability issue described herein is inherent to any age-specific cancer incidence data that is linear at older ages. This way, such problems can be anticipated and handled systematically, e.g. by reparameterizing the model appropriately. In theory, we could gain additional information if people were to live long enough to see the incidence plateau, but, as previously discussed, the expected timing of the plateau in the three- and four-stage clonal expansion models is well beyond conceivable human life spans. While the observation of a plateau might suggest that either the underlying mechanism is the two-stage model or the presence of heterogeneities or temporal effects, the absence of a plateau leaves room for various interpretations. Indeed, the two-, three-, and four-stage models were all able to reasonably fit the pancreatic cancer incidence data (Fig 3).

For each of the two-, three-, and four-stage models, only three parameter combinations were practically identifiable. In each case, these combinations are most easily interpreted in the following forms:

$$\alpha -\beta -\mu_{n-1}$$ (12)

the net cell proliferation rate of initiated cells,

$$\alpha \mu_{n-1}$$ (13)

the scaled malignant conversion rate, and

$$\nu \;\left(\prod_{i=1}^{n-2}\mu_{i}\right)\;\left(X/\alpha \right)$$ (14)

the product of all preinitation rates scaled by the number of normal cells and the cell growth rate. Note that the first two combinations are together equivalent to *p*_*n*_ and *q*_*n*_. Because the last combination is a product of individually structurally identifiable combinations, we know that information about mutation rates at the intermediate steps is only available in later features of the MSCE hazards, i.e. the asymptote and the transition from the linear phase to the asymptote.

Because there are only three practically identifiable combinations, successful parameter estimation can only be achieved if the models are reparameterized in terms of these combinations. For example, with the three-stage model parameterized as in Eq (3), parameter estimates for *νX* and *μ*_1_/*α* are not stable. Here, we proposed one possible solution with the reparameterizations in Eqs (9) and (11) and show that it does indeed resolve the practical unidentifiabilities, though an infinite number of reparameterizations will give equivalent fits as long as the parameter combinations are preserved. Each reparameterization represents a different assumption about the relative sizes of its constituent parameter combinations. Our reparameterizations are inspired by the assumption that the preinitation mutation rates are equal (*ν* = *μ*_1_ = …) but do not actually codify this assumption in the models. Nevertheless, it is consistent with a scenario in which multiple copies of a tumor suppressor gene must be “knocked out” [1].

Traditional approaches to parameter estimation that use asymptotic confidence intervals do not always reveal practical identifiability issues. Because asymptotic confidence intervals are based on the local curvature of the likelihood around the parameter estimate, they may give finite confidence bounds when the likelihood is curved on one side of the estimate but flat on the other. Numerical optimization algorithms may provide results that give the appearance of practical identifiability but have in fact simply pushed the estimate to the point where the likelihood begins to curve. Hence, the fact that our group and others have previously reported values of *μ*_2_/*α* with finite confidence intervals in four-stage models [6, 8] is not inconsistent with our results. Some of these previous works have interpreted the larger-than-expected values for *μ*_2_ (fixing *α*) as being too fast to represent a genetic mutation, suggesting that the four-stage model may represent two, slow genetic mutations followed by a fast epigenetic change, a transient event, or other transformation. Our results suggest that a large range of values *μ*_2_/*α* would have resulted in equivalent fits, and we note that the values presented in these previous works are of the same order of magnitude where we see curvature in our likelihood function. In particular, a previous fit of pancreatic cancer incidence in SEER (1973–2004) using the four-stage model [8] estimated *νXμ*_1_*μ*_2_/*α* to be 1.88E-5—the same value that we find here with the new parametrization (for pancreatic cancer in SEER 1973–2012; Table 1)—but also separately estimated *μ*_2_/*α* to be 4.0E-1, which falls exactly where the profile likelihood begins to curve up (Fig 8). Hence, such parameter estimates may be an artifact of the algorithm numerically optimizing the likelihood, and one should then be careful when giving a biological interpretation to those results.

This analysis also speaks to the question of model selection and model reduction. Although the four-stage model gives the best statistical fit to the data in Fig 3, its hazard nearly entirely overlaps with the that of the other models. Hence, we must question whether or not the larger model is actually capturing some nuance in the data. Given the practical identifiability issues we have presented, does the two-stage already capture all of the information? Possibly so. Are the results of both models equivalent? Unfortunately not: although each model is estimating the same biological parameters (i.e. the product of initiation rates, the final promotion rate, and the malignant conversion rate), a perusal of Table 1 reveals that the parameter estimates are not particularly consistent across the three models (although are generally within an order of magnitude). Moreover, the different dynamics of each model will become important as we move away from simply analyzing incidence and consider prediction or individual time-varying exposures. Nevertheless, in this situation, one might be inclined to take an ensemble approach and to consider uncertainty quantification not only within a model but across the models, perhaps weighting in some way by statistical fit. Additional empirical science, by better elucidating carcinogenesis mechanisms common to cancer at given site, could aid modelers in model selection.

The guidance we have presented in this study is important as three- and four-stage clonal expansion models are commonly used to model certain cancers at the population level, and successful parameter estimation is dependent on the model being identifiable with respect to the available data. Ultimately, our analysis demonstrates the need for future studies to verify the practical identifiability of model parameters whenever feasible, which should strengthen the validity of the analyses and aid in the interpretation of estimated parameter values and modeling results.

## Supporting information

S1 Data Incidence. Cases of pancreatic cancer in men reported to SEER 9, 1973–2012.
(CSV)

S2 Data Population. Population of men in SEER 9 catchment, 1973–2012.
(CSV)

S1 Text Mathematical and statistical details. Derivation of multistage clonal expansion model hazards and statistical formulation of the likelihood.
(PDF)
