# Correction: Parameter estimation for multistage clonal expansion models from cancer incidence data: A practical identifiability analysis

**DOI:** 10.1371/journal.pcbi.1005660

**Published:** 2017-07-17

**Authors:** Andrew F. Brouwer, Rafael Meza, Marisa C. Eisenberg

Table 1 and Figs 4, 5, 7, 8 and 10 are incorrect. There is an error in the value of the threshold used to calculate the confidence intervals. The authors have provided the corrected versions here.

**Table 1:** Best-fit parameters and likelihood-based 95% confidence intervals. Best-fit parameters and likelihood-based 95% confidence intervals for the fits of the two-, three-, and four-stage clonal expansion models (with parameterizations using only practically identifiable parameter combinations and given in Eqs. (2), (9), and (11) respectively) to age-specific incidence of pancreatic cancer.

Model	Parameter combination	Value	Likelihood-based 95% CI
Two-stage			
	$p_{2}=\frac{1}{2}\left(-\left(\alpha -\beta -\mu_{1}\right)-\sqrt{{\left(\alpha -\beta -\mu_{1}\right)}^{2}-4\alpha \mu_{1}}\right)$	-1.29E-1	(-1.32E-1, -1.26E-1)
	$q_{2}=\frac{1}{2}\left(-\left(\alpha -\beta -\mu_{1}\right)+\sqrt{{\left(\alpha -\beta -\mu_{1}\right)}^{2}-4\alpha \mu_{1}}\right)$	6.21E-6	(5.58E-6,6.88E-6)
	*r*_2_ = *vX*/*α*	1.50E-2	(1.38E-2,1.65E-2)
Three-stage			
	$p_{3}=\frac{1}{2}\left(-\left(\alpha -\beta -\mu_{2}\right)-\sqrt{{\left(\alpha -\beta -\mu_{2}\right)}^{2}-4\alpha \mu_{2}}\right)$	-1.38E-1	(-1.42E-1,-1.34E-1)
	$q_{3}=\frac{1}{2}\left(-\left(\alpha -\beta -\mu_{2}\right)+\sqrt{{\left(\alpha -\beta -\mu_{2}\right)}^{2}-4\alpha \mu_{2}}\right)$	1.57E-5	(1.38E-5,1.76E-5)
	$r_{3}=\sqrt{\nu X\mu_{1}/\alpha }$	2.35E-2	(2.23E-2,2.49E-2)
Four-stage			
	$p_{4}=\frac{1}{2}\left(-\left(\alpha -\beta -\mu_{3}\right)-\sqrt{{\left(\alpha -\beta -\mu_{3}\right)}^{2}-4\alpha \mu_{3}}\right)$	-1.50E-1	(-1.57E-1,-1.43E-1)
	$q_{4}=\frac{1}{2}\left(-\left(\alpha -\beta -\mu_{3}\right)+\sqrt{{\left(\alpha -\beta -\mu_{3}\right)}^{2}-4\alpha \mu_{3}}\right)$	4.59E-5	(3.90E-5,5.32E-5)
	*r*_3_ = (*vXμ*_1_*μ*_2_*/α*)^1/3^	2.66E-2	(2.54E-2,2.80E-2)

**Fig 4:**
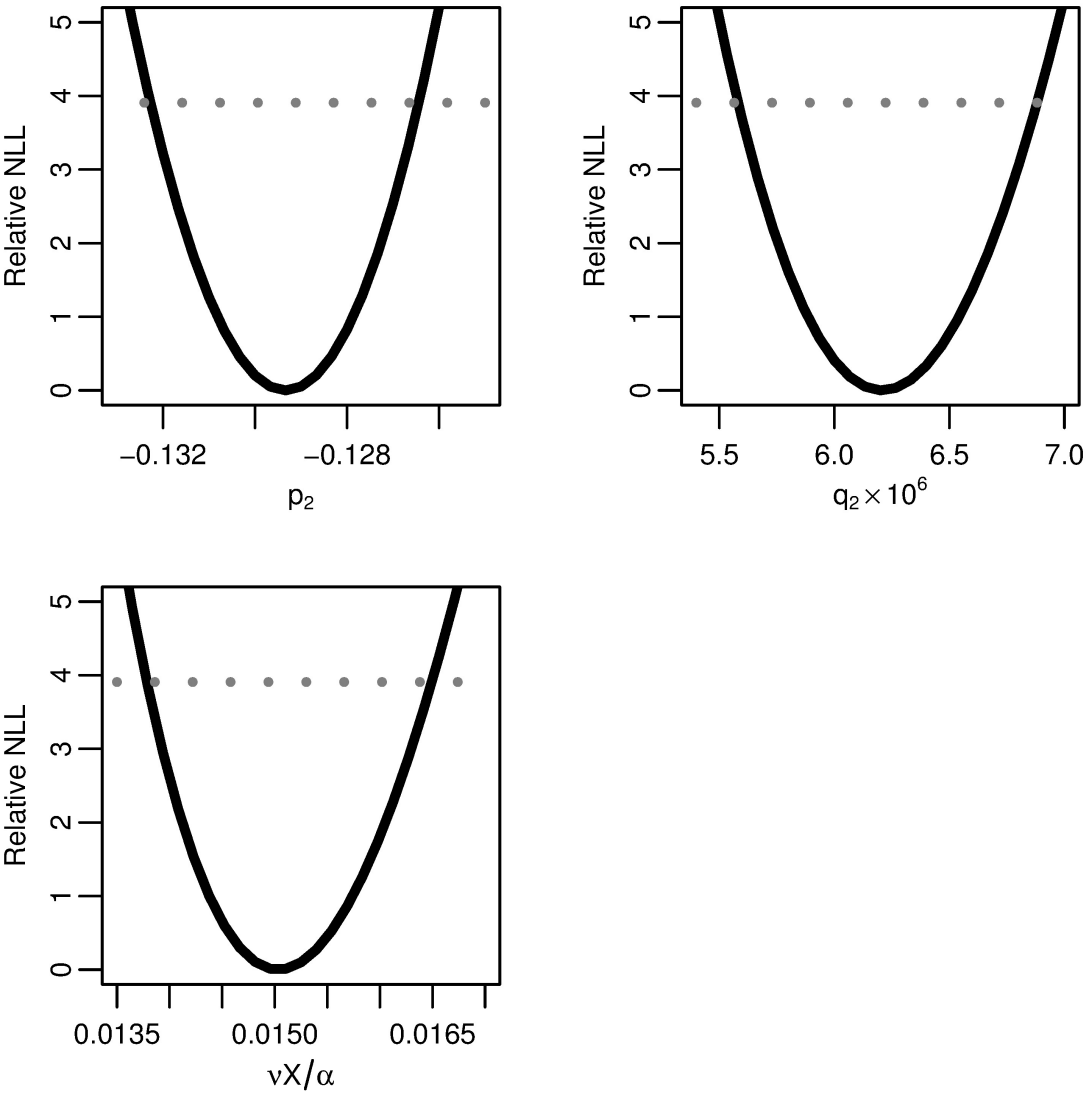
Two-stage model profile likelihoods. Profiles of the relative negative log-likelihood (NLL) of the two-stage clonal expansion model as each of the parameter combinations *p*_2_, *q*_2_, and *νX/α* are varied while the remaining parameters are fit. The gray dotted line gives the *α* = 0.05 threshold for simultaneous confidence intervals based on the relative negative log-likelihood. All three parameters are identifiable.

**Fig 5:**
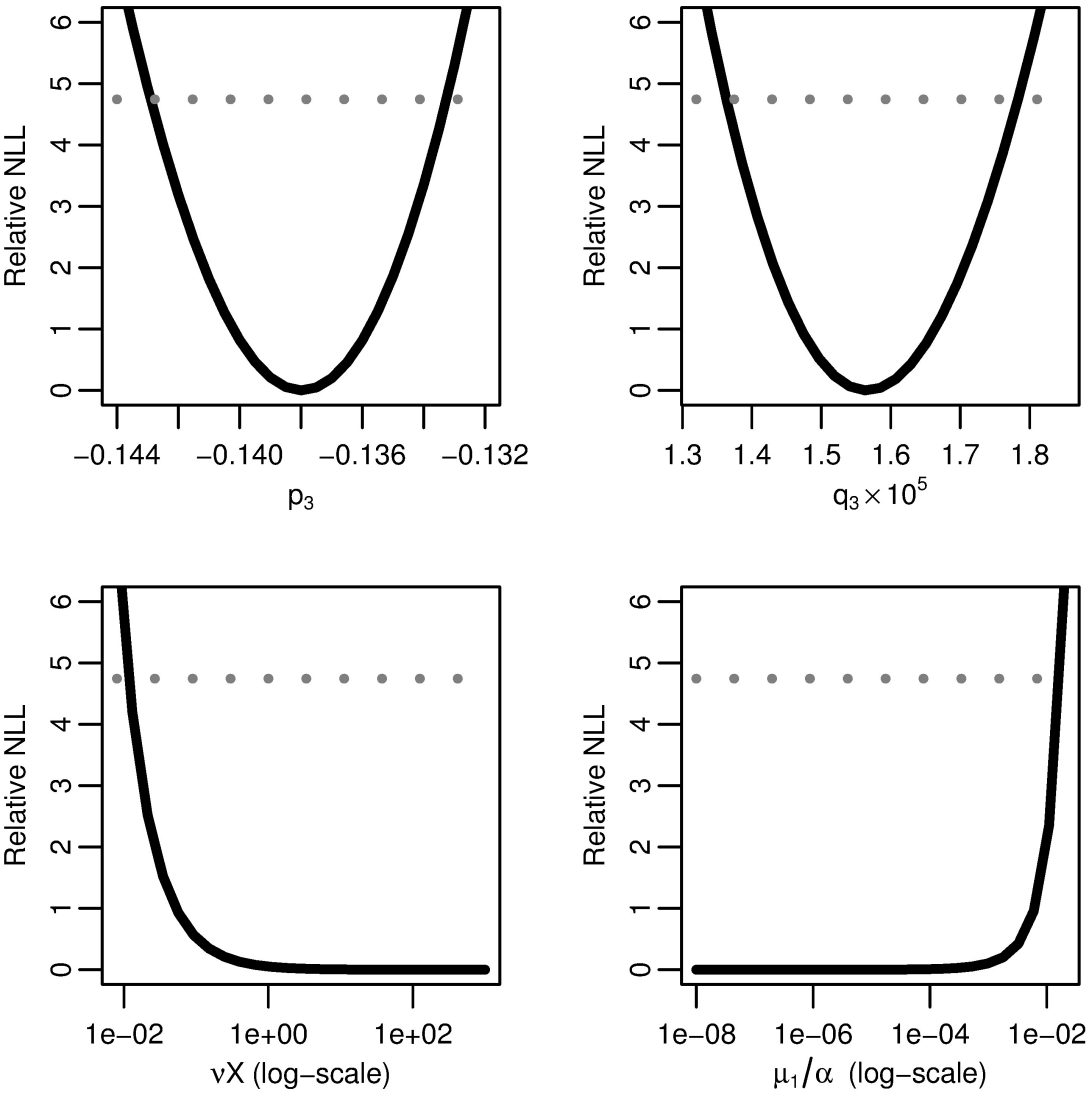
Three-stage model profile likelihoods. Profiles of the relative negative log-likelihood (NLL) of the three-stage clonal expansion model as each of parameter combinations *p*_3_, *q*_3_, *νX*, and *μ*_1_*/α* are varied while the remaining parameters are fit. The gray dotted line gives the *α* = 0.05 threshold for simultaneous confidence intervals based on the relative negative log-likelihood. Parameter combinations *p*_3_ and *p*_4_ are identifiable, while *νX* and *μ*_1_*/α* are practically unidentifiable.

**Fig 7:**
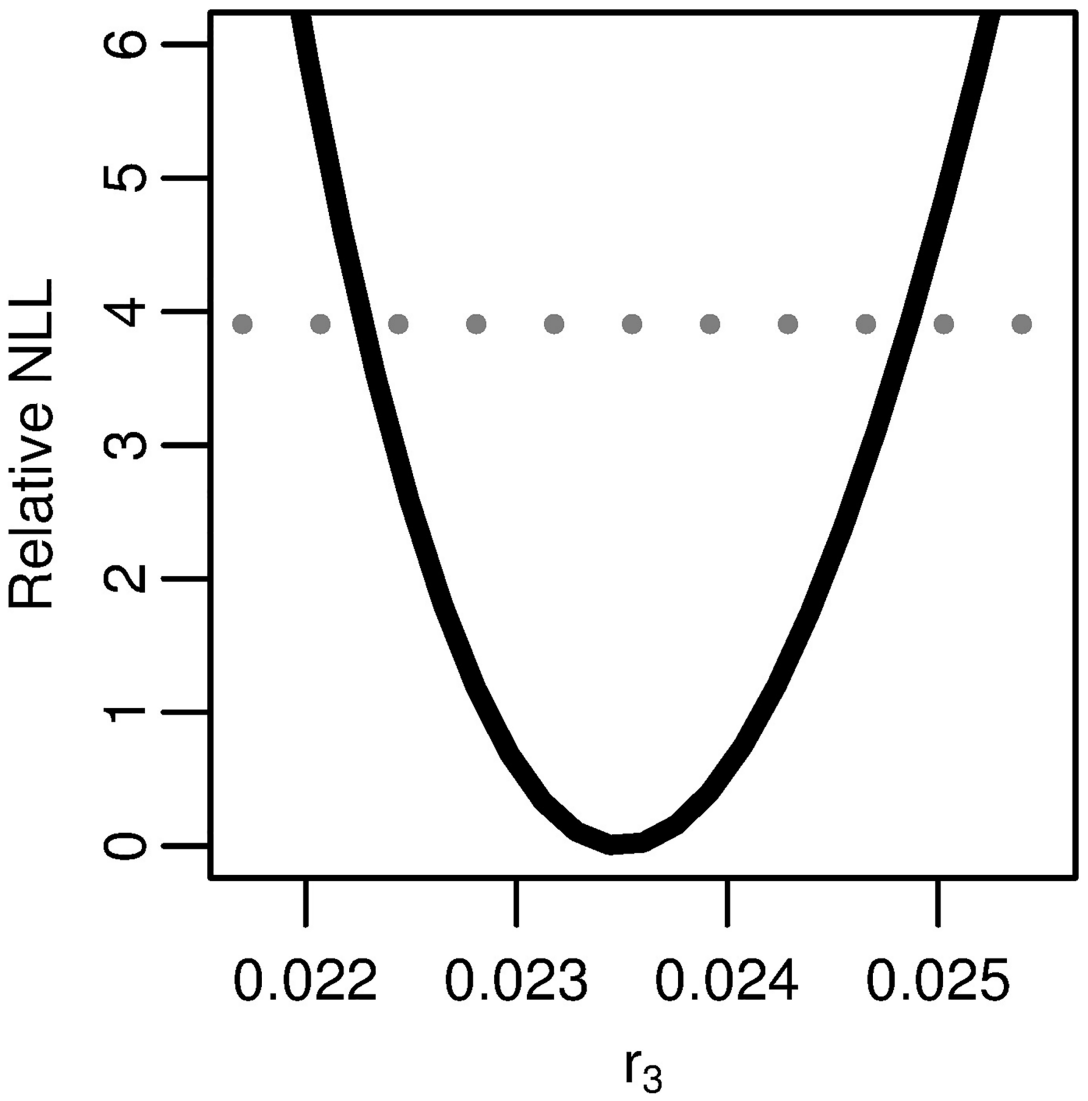
Profile likelihood for the reparameterized combination of the three-stage model. Profile of the relative negative log-likelihood (NLL) as the parameter $r_{3}=\sqrt{\nu X\mu 1/\alpha }$ varied while the remaining parameters are fit in the three-stage clonal expansion model. The gray dotted line gives the *α* = 0.05 threshold for simultaneous confidence intervals based on the relative negative log-likelihood. Parameter combination *r*_3_ is identifiable.

**Fig 8:**
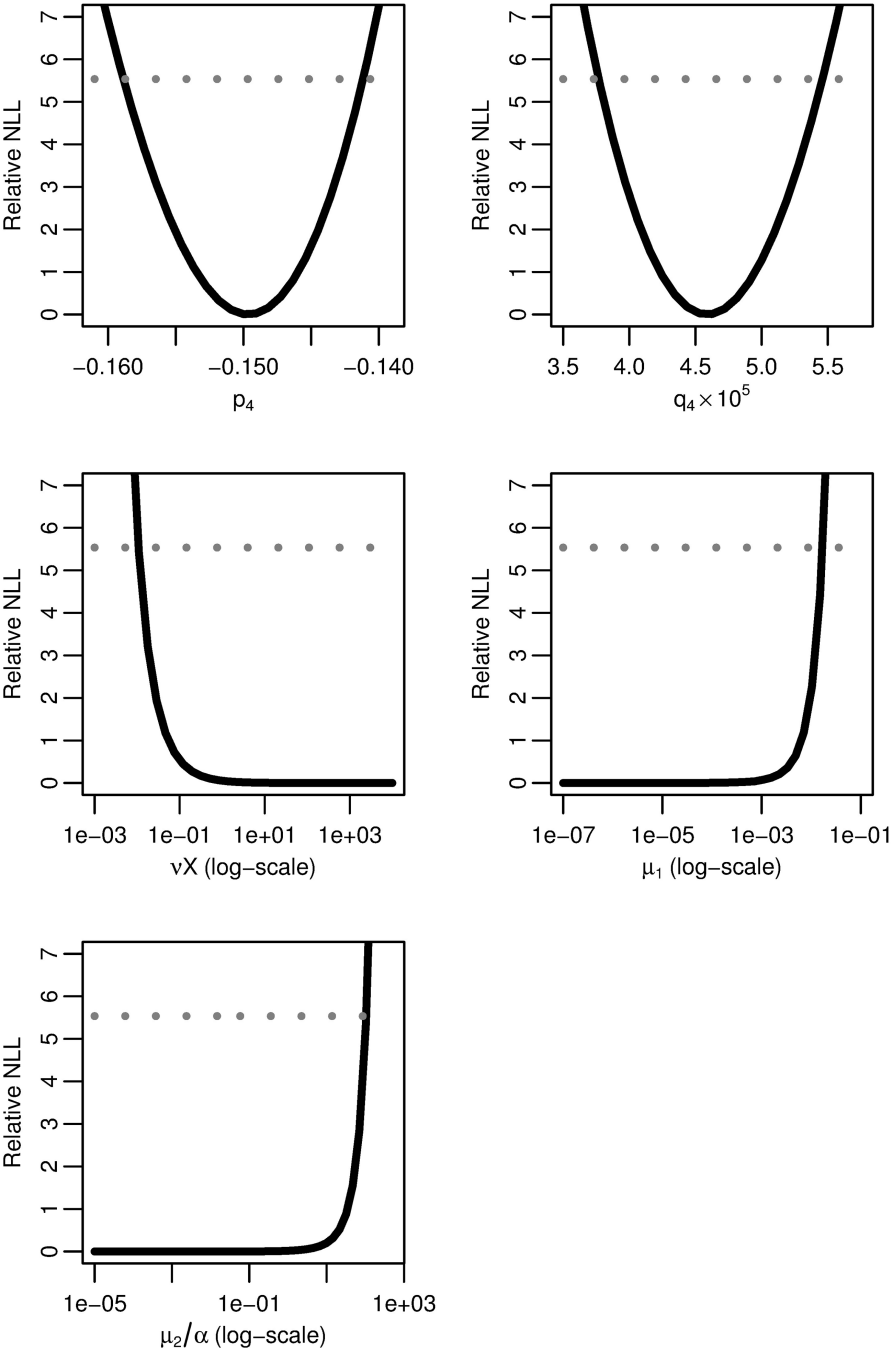
Four-stage model profile likelihoods. Profiles of the relative negative log-likelihood (NLL) of the four-stage clonal expansion model as each of parameter combinations *p*_4_, *q*_4_, *νX*, *μ*_1_, and *μ*_2_*/α* are varied while the remaining parameters are fit. The gray dotted line gives the *α* = 0.05 threshold for simultaneous confidence intervals based on the relative negative log-likelihood. Parameter combinations *p*_4_ and *q*_4_ are identifiable, while *νX*, *μ*_1_, and *μ*_2_*/α* are practically unidentifiable.

**Fig 10:**
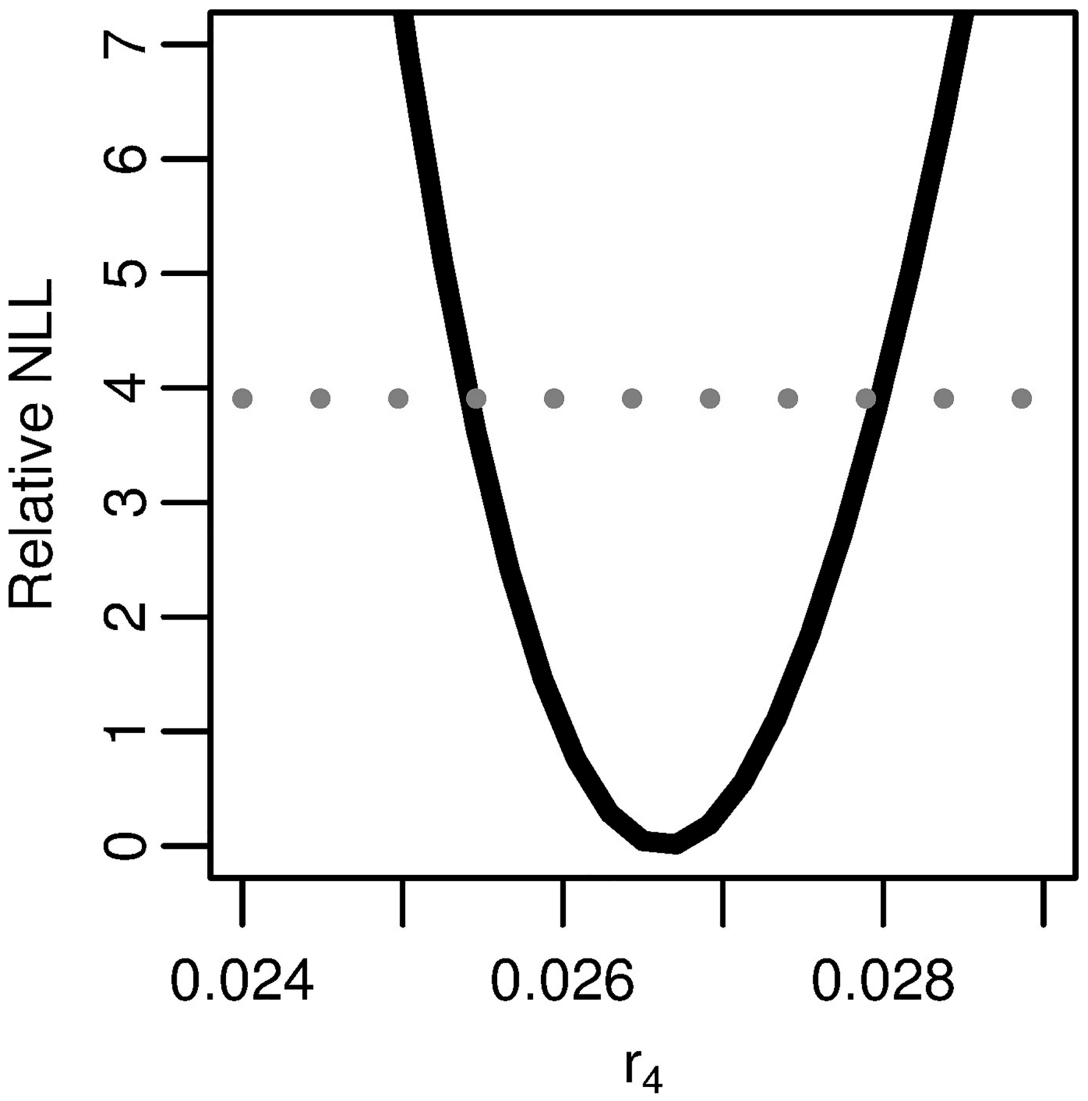
Profile likelihood for the reparameterized combination of the four-stage model. Profile of the relative negative log-likelihood (NLL) as the parameter *r*_4_ = (*νXμ*_1_*μ*_2_*/α*)^1/3^ is varied while the remaining parameters are fit in the four stage clonal expansion model. The gray dotted line gives the *α* = 0.05 threshold for simultaneous confidence intervals based on the relative negative log-likelihood. Parameter combination *r*_4_ is identifiable.
