# Supplementary material for: Parameter estimation for multistage clonal expansion models from cancer incidence data: A practical identifiability analysis
Source: PLoS Comput Biol. 2017 Mar 13;13(3):e1005431. doi: 10.1371/journal.pcbi.1005431 (PMC5367820; doi:10.1371/journal.pcbi.1005431)
Supplement: S1 Text — Derivation of multistage clonal expansion model hazards and statistical formulation of the likelihood. (PDF) [file pcbi.1005431.s003.pdf]

Supporting Information for *Parameter estimation for multistage clonal expansion models from cancer incidence data: a practical identifiability analysis*

Multistage clonal expansion model hazard derivation

The derivation of the multistage clonal expansion model hazards has been previously shown (e.g. in the appendices of Luebeck and Moolgavkar (2002) and Jeon (2007).) We reproduce it here with our notation.

The n-stage clonal expansion model is a continuous-time Markov chain with the following states: $X(t)$, the number of normal cells at age t; $Y_1(t), \ldots, Y_{n-2}$, the number of cells in subsequent preintiation states; $Y_{n-1}(t)$, the number of initiated cells; and $Z(t)$, the number of malignant cells. Let ν be the initial mutation rate (with $\mu_0 := \nu X$), $\mu_1, \ldots, \mu_{n-3}$ the following preinitiation mutation rates, μ_{n-2} the initiation mutation rate, μ_{n-1} the malignant transformation rate, α the clonal expansion rate, and β the cell death rate. Mutations are modeled as an asymmetric division process resulting in one cell of the same class as the parent and one of the subsequent class. For notational simplicity, we suppress the possible dependence of the parameters on time.

Let

$$P_{(j_1,\ldots,j_{n-1},k)}(t) = P\left[Y_1(t) = j_1, \ldots, Y_{n-1}(t) = j_{n-1}, Z(t) = k | Y_1(0) = 0, \ldots, Y_{n-1}(0) = 0, Z(0) = 0\right]. \tag{S1}$$

Then, the probability generating function for the numbers of preinitiated, initiated, and malignant cells may be written as

$$\Psi(y_1, \ldots, y_{n-1}, z, t) = E\left[y_1^{Y_1(t)} \cdots y_{n-1}^{Y_{n-1}(t)} z^{Z(t)} | Y_1(0) = 0, \ldots, Y_{n-1}(0) = 0, Z(0) = 0\right], \tag{S2}$$

$$= \sum_{(j_1,\ldots,j_{n-1},k)} P_{(j_1,\ldots,j_{n-1},k)}(t) y_1^{j_1} \cdots y_{n-1}^{j_{n-1}} z^k. \tag{S3}$$

We wish to solve for the survival and hazard functions,

$$S_n(t) = \sum_{(j_1,\ldots,j_{n-1},0)} P_{(j_1,\ldots,j_{n-1},0)}(t), \tag{S4}$$

$$= \sum_{(j_1,\ldots,j_{n-1},k)} P_{(j_1,\ldots,j_{n-1},k}(t) 1^{j_1} \cdots 1^{j_{n-1}} 0^k, \tag{S5}$$

$$= \Psi(1, \ldots, 1, 0, t), \tag{S6}$$

$$h_n(t) = -\frac{d}{dt} \ln \Psi \Big|_{(1,\ldots,1,0,t)}. \tag{S7}$$

To this end, we write the Kolmogorov forward equations for the transition probabilities of this

process,

$$
\begin{aligned}
\frac{d}{dt}P_{(j_1,\ldots,j_{n-1},k)} &= \mu_0 P_{(j_1-1,\ldots,j_{n-1},k)} \\
&+ j_1\mu_1 P_{(j_1,j_2-1,\ldots,j_{n-1},k)} + \cdots + j_{n-2}\mu_{n-2} P_{(j_1,\ldots,j_{n-2},j_{n-1}-1,k)} \\
&+ j_{n-1}\mu_{n-1} P_{(j_1,\ldots,j_{n-1},k-1)} \\
&+ \alpha(j_{n-1}-1) P_{(j_1,\ldots,j_{n-1}-1,k)} + \beta(j_{n-1}+1) P_{(j_1,\ldots,j_{n-1}+1,k)} \\
&- \left(\mu_0 + j_1\mu_1 + \cdots + j_{n-2}\mu_2 + j_{n-1}(\alpha+\beta+\mu_{n-1})\right) P_{(j_1,\ldots,j_{n-1},k)}.
\end{aligned}
\tag{S8}
$$

Multiplying both sides by $y_1^{j_1}\ldots y_{n-1}^{j_{n-1}} z^k$ and summing over all $(j_1,\ldots,j_{n-1},k)$, we arrive at the forward Kolmogorov equation for the probability generating function,

$$
\begin{aligned}
\frac{\partial\Psi}{\partial t} &= (y_1-1)\mu_0\Psi \\
&+ (y_2-1)\mu_1 y_1 \frac{\partial\Psi}{\partial y_1} + \cdots + (y_{n-1}-1)\mu_{n-2} y_{n-2} \frac{\partial\Psi}{\partial y_{n-1}} \\
&+ \left(\alpha y_{n-1}^2 + \beta - (\alpha+\beta+\mu_{n-1}(1-z)) y_{n-1}\right) \frac{\partial\Psi}{\partial y_{n-1}}.
\end{aligned}
\tag{S9}
$$

Because all cells are assumed to be normal at time $t=0$, $\Psi=1$ at this time. The partial differential equation in Eq. (S9) is first-order and thus may be solved by the method of characteristics. We choose characteristic curves $(y_1(\tau),\ldots,y_{n-1}(\tau),z(\tau),t(\tau))$ such that the following ordinary differential equations are satisfied:

$$
\begin{aligned}
\frac{dy_1}{d\tau} &= (1-y_2)\mu_1 y_1, \\
&\vdots \\
\frac{dy_{n-2}}{d\tau} &= (1-y_{n-1})\mu_{n-2} y_{n-2}, \\
\frac{dy_{n-1}}{d\tau} &= (\alpha+\beta+\mu_{n-1}(1-z)) y_{n-1} - \alpha y_{n-1}^2 - \beta, \\
\frac{dz}{d\tau} &= 0, \\
\frac{dt}{d\tau} &= 1.
\end{aligned}
\tag{S10}
$$

We then write Eq. (S9) as an ordinary differential equation,

$$
\frac{d\Psi}{d\tau} = (y_1(\tau)-1)\mu_0\Psi, \tag{S11}
$$

which has the solution

$$
\Psi(\tau) = \exp\left[\int_{\tau_0}^{\tau} (y_1(u)-1)\mu_0\, du\right], \tag{S12}
$$

where τ_0 is such that $t(\tau_0)=0$, so that $\Psi(\tau_0)=1$ by our initial condition.

Because this equation depends on y_1, we must further consider the system of equations in Eqs. (S10). Because we are solving for $S(t) = \Psi(1, \ldots, 1, 0, t)$, we consider the characteristic curve, that is $(y_1(\tau), \ldots, y_{n-1}(\tau), z(\tau), t(\tau))$, with boundary condition $(1, \ldots, 1, 0, t^*)$, where t^* denotes a fixed t. Along this characteristic, $t = \tau - \tau_0$, $z = 0$, and y_{n-1} satisfies a Ricatti equation (Heidenreich et al., 1997), which, in the case of constant parameters, has the solution (with $\tau^* = \tau_0 + t^*$)

$$y_{n-1}(\tau) = \frac{p_n q_n}{\alpha}\left(\frac{e^{-p_n(\tau^*-\tau)} - e^{-q_n(\tau^*-\tau)}}{q_n e^{-p_n(\tau^*-\tau)} - p_n e^{-q_n(\tau^*-\tau)}}\right) + 1, \tag{S13}$$

where

$$p_n, q_n := \frac{1}{2}\left(-(\alpha - \beta - \mu_{n-1}) \mp \sqrt{(\alpha - \beta - \mu_{n-1})^2 + 4\alpha\mu_{n-1}}\right). \tag{S14}$$

Moreover, each $y_i(\tau)$, for $1 \leq i \leq n-2$, can be found recursively, noting $y_i(\tau^*) = 1$,

$$y_i(\tau) = \exp\left[\int_{\tau^*}^{\tau} (1 - y_{i+1}(u))\mu_i \, du\right]. \tag{S15}$$

Although the backward formulation (see, for example, Brouwer et al. (2016)) is more computationally tractable, this forward formulation allows us to find formulas for the hazards of the first several models in the constant parameter case.

Two-stage model

For the two-stage model, we can analytically derive the solution for Ψ in the constant parameter case.

$$\Psi(\tau) = \exp\left[\int_{\tau_0}^{\tau} (y_1(u) - 1)\mu_0 \, du\right], \tag{S16}$$

$$= \exp\left[\int_{\tau_0}^{\tau} \left(\frac{p_2 q_2}{\alpha}\left(\frac{e^{-p_2(\tau^*-u)} - e^{-q_2(\tau^*-u)}}{q_2 e^{-p_2(\tau^*-u)} - p_2 e^{-q_2(\tau^*-u)}}\right)\right)\mu_0 \, du\right], \tag{S17}$$

$$= \left(\frac{q_2 e^{-p_2(\tau^*-\tau)} - p_2 e^{-q_2(\tau^*-\tau)}}{q_2 e^{-p_2(\tau^*-\tau_0)} - p_2 e^{-q_2(\tau^*-\tau_0)}}\right)^{\frac{\mu_0}{\alpha}}. \tag{S18}$$

Expressed as a function of t,

$$\Psi(t) = \left(\frac{q_2 e^{-p_2(t^*-t)} - p e^{-q_2(t^*-t)}}{q_2 e^{-p_2 t^*} - p_2 e^{-q_2 t^*}}\right)^{\frac{\mu_0}{\alpha}}. \tag{S19}$$

Since t^* is the value of t such that $(y_1, \ldots, y_{n-1}, z) = (1, \ldots, 1, 0)$, $S(t^*) = \Psi(t^*)$. Viewing S as a function of $t = t^*$,

$$S_2(t) = \left(\frac{q_2 - p_2}{q_2 e^{-p_2 t} - p_2 e^{-q_2 t}}\right)^{\frac{\mu_0}{\alpha}}, \tag{S20}$$

and

$$h_2(t) = \frac{\mu_0}{\alpha}\left(\frac{p_2 q_2 \left(e^{-q_2 t} - e^{-p_2 t}\right)}{q_2 e^{-p_2 t} - p_2 e^{-q_2 t}}\right). \tag{S21}$$

Three-stage model

For the three-stage model, we have

$$\Psi(\tau) = \exp\left[\int_{\tau_0}^{\tau} (y_1(u_1) - 1)\mu_0 \, du_1\right], \tag{S22}$$

$$= \exp\left[\int_{\tau_0}^{\tau} \left(\exp\left[\int_{\tau^*}^{u_1} (1 - y_2(u_2))\mu_1 \, du_2\right] - 1\right) \mu_0 \, du\right], \tag{S23}$$

$$= \exp\left[\int_{\tau_0}^{\tau} \left(\exp\left[\int_{\tau^*}^{u_1} \left(-\frac{p_3 q_3}{\alpha}\left(\frac{e^{-p_3(\tau^*-u_2)} - e^{-q_3(\tau^*-u_2)}}{q_3 e^{-p_3(\tau^*-u_2)} - p_3 e^{-q_3(\tau^*-u_2)}}\right)\right) \mu_1 \, du_2\right] - 1\right) \mu_0 \, du_1\right], \tag{S24}$$

$$= \exp\left[\int_{\tau_0}^{\tau} \left(\left(\frac{q_3 - p_3}{q_3 e^{-p_3(\tau^*-u_1)} - p_3 e^{-q_3(\tau^*-u_1))}}\right)^{\frac{\mu_1}{\alpha}} - 1\right) \mu_0 \, du_1\right], \tag{S25}$$

and

$$S_3(t) = \exp\left[\int_0^t \left(\left(\frac{q_3 - p_3}{q_3 e^{-p_3(t-u)} - p_3 e^{-q_3(t-u))}}\right)^{\frac{\mu_1}{\alpha}} - 1\right) \mu_0 \, du\right]. \tag{S26}$$

Although this survival function cannot be written in closed form, the hazard can be,

$$h_3 = -\frac{d}{dt} \ln S_3(t), \tag{S27}$$

$$= \mu_0 \left(1 - \left(\frac{q_3 - p_3}{q_3 e^{-p_3 t} - p_3 e^{-q_3 t}}\right)^{\frac{\mu_1}{\alpha}}\right). \tag{S28}$$

Four-stage model

For the four-stage model, we continue to describe Ψ recursively,

$$\Psi(\tau) = \exp\left[\int_{\tau_0}^{\tau} \left(\exp\left[\int_{\tau^*}^{u_1} \left((1 - \left(\exp\left[\int_{\tau^*}^{u_2} (1 - y_3(u_3))\mu_2 \, du_3\right]\right)\right) \mu_1 \, du_2\right] - 1\right) \mu_0 \, du\right], \tag{S29}$$

which leads to the following integral formula for the hazard

$$h_4(t) = \nu X \left(1 - \exp\left(\int_0^t \mu_1 \left(\left(\frac{q_4 - p_4}{q_4 e^{-p_4(t-u)} - p_4 e^{-q_4(t-u)}}\right)^{\mu_2/\alpha} - 1\right) du\right)\right). \tag{S30}$$

Model likelihood

We assume that incidence of cancer is Poisson-distributed with mean number of cases

$$\mu_i = N_i \cdot h_n(\boldsymbol{\rho}_n, t_i) \tag{S31}$$

at age t_i with population N_i; here, $n \in \{2, 3, 4\}$, and $\boldsymbol{\rho}_n$ is $\{p_2, q_2, \nu X/\alpha\}$, $\{p_3, q_3, \nu X, \mu_1/\alpha\}$, and $\{p_4, q_4, \nu X, \mu_1, \mu_2/\alpha\}$, respectively. The likelihood can then be written as

$$\mathcal{L}(\boldsymbol{\rho}_n) = \prod_i \frac{e^{-\mu_i} \mu_i^{x_i}}{x_i!} \tag{S32}$$

where $\{x_i\}$ are the numbers of observed cases. Maximizing the likelihood function—as a function of the parameters comprising $\boldsymbol{\rho}_n$—is equivalent to minimizing the negative log-likelihood (NLL)

$$\mathrm{NLL}(\boldsymbol{\rho}_n) = -\sum_i \left(-\mu_i + x_i \log \mu_i - \log x_i!\right). \tag{S33}$$
